# Supplementary material for: Interplay between oceanic subduction and continental collision in building continental crust
Source: Nat Commun. 2022 Nov 21;13:7141. doi: 10.1038/s41467-022-34826-0 (PMC9681875; doi:10.1038/s41467-022-34826-0)
Supplement: Supplementary file 1 — Supplementary Information [file 41467_2022_34826_MOESM1_ESM.pdf]

Supplementary Information

Interplay between oceanic subduction and continental collision in building continental crust

Di-Cheng Zhu, Qing Wang, Roberto F. Weinberg, Peter A. Cawood, Sun-Lin Chung, Yong-Fei Zheng, Zhi-Dan Zhao, Zeng-Qian Hou, and Xuan-Xue Mo

Inventory of Supporting Information:

Supplementary Methods

- Zircon LA–ICP–MS U–Pb dating
- SIMS oxygen isotope analyses of zircons
- Electron-probe microanalyses
- Identifying the subducted sediment from the pre-existing basement

Supplementary Figures

- **Supplementary Fig. 1** Spatial and temporal distributions of ultramafic rocks (SiO_2 < 45 wt%) in the Gangdese belt
- **Supplementary Fig. 2** Distributions of K_2O contents of samples from continental and island arcs
- **Supplementary Fig. 3** Zircon evidence for the re-melting of older crustal rocks in the Gangdese belt

Supplementary Methods

Zircon LA–ICP–MS U–Pb dating

Zircon LA–ICP–MS U–Pb dating was conducted at the Mineral Laser Microprobe Analysis Laboratory (Milma Lab), China University of Geosciences, Beijing (CUGB). Laser sampling was performed using a NewWave 193UC excimer laser ablation system. The ablated material was transported by carrier gas into the plasma source of an Agilent 7900 ICP–MS. Detailed setting parameters for the instruments and experimental process are provided in ref. 1.

Off-line selection and integration of background and analyte signals, and time-drift correction and quantitative calibration for trace element analyses and U–Pb dating were performed using ICPMSDataCal software. Common Pb corrections were calculated using ComPbCorr#3.17 and concordia diagrams, and $^{206}Pb/^{238}U$ weighted mean plots were made using Isoplot. Zircon standards GJ-1 and Plesovice were analyzed as unknown samples that were inserted between 91500 and the samples. We obtained weighted mean $^{206}Pb/^{238}U$ ages of 601.5 ± 4.3 Ma (2SD, n = 42) for GJ-1 and 336.3 ± 2.9 Ma (2SD, n = 42) for Plesovice, which are within error of recommended values[2,3]. Data are given in **Supplementary Table 2**.

Electron-probe microanalyses

Based on petrographic observations, mineral compositions were determined using a EPMA1720 electron microprobe at the EPMA Lab, China University of Geosciences, Beijing. The analyses were performed in a wavelength dispersive mode under the condition of an acceleration voltage of 15 kV, beam current of 10 nA and focused beam width of 1−2 μm. The peak counting time and background duration were 10−30 s and 10 s.

Natural minerals (Mineral Standard Mount NINM25-53, Astimex Scientific) were adopted for calibration different elements: olivine for Si and Mg, rutile for Ti, garnet for Fe, rhodonite for Mn, diopside for Ca, pentlandite for Ni, chromite for Cr and Al, and willemite for Zn. The intensity data were corrected using the ZAF3 on-line analytical procedure. Relative errors are <1% for major elements in olivine (Si, Mg, and Fe) and spinel (Al, Mg, Cr, and Fe) and <15% for other minor elements. Data are given in **Supplementary Table 3**.

SIMS oxygen isotope analyses of zircons

The mounts of zircons were polished and coated with gold for *in situ* SIMS analysis. Oxygen isotope analyses of zircons were performed using a CAMECA IMS-1280 multi-collector ion probe at IGGCAS, using the procedure described by previous works[4,5]. The spot size was ~20 μm (10 μm beam diameter + 10 μm raster). Secondary ions were extracted at a −10 kV potential. Oxygen isotopes were measured using a multi-collection mode and the mass resolution used to measure oxygen isotopes was 2500.

Measured $^{18}O/^{16}O$ ratios were normalized to Vienna Standard Mean Ocean Water compositions (VSMOW; $^{18}O/^{16}O$ = 0.0020052), and then corrected for instrumental mass fractionation (IMF) using the Penglai zircon standard. Measurement of the in-house standard Qinghu during the session yielded a value of 5.42 ± 0.20‰ (2SD, n = 38), which is identical to the recommended value of 5.4 ± 0.2‰ (2SD)[4]. Data are given in **Supplementary Table 4**.

Identifying the subducted sediment from the pre-existing basement

Distinguishing the subducted sediment from the pre-existing overriding basement (i.e. the upper plate) has been a significant challenge. However, it remains possible using mixing modelling of well constrained end members[6-8]. This is because the curvature of mixing curves, and thus the amounts of each end member, are controlled by the relative abundances of trace elements of the selected end members. It has been established that zircon $\delta^{18}O$ remain unaffected by fractional crystallization and variable temperature[9]. This makes the zircon $\delta^{18}O$ an effective monitor of the involvement of recycled pre-existing crustal materials with high and variable $\delta^{18}O$ due to low-temperature alteration[9,10]. In our study, to distinguish the subducted sediment from the pre-existing Gangdese basement, we use the Hf abundances and Hf-O isotopic compositions of each local end member as introduced below.

Neo-Tethys altered oceanic crust is represented by diabase, gabbro, and basalt from the Xigaze ophiolite within the Yarlung Zangbo suture zone[11].

Neo-Tethys subducted oceanic sediment is taken from ref. 12 for the ε_{Hf} values and Hf abundances, and from ref. 9 for the zircon $\delta^{18}O$ values. The exact amounts of

sediment contributions are difficult to constrain because (1) the ε_{Hf} values and Hf abundances are not available for the Neo-Tethys oceanic sediments and (2) the global subducted oceanic sediments display large variation of $\delta^{18}O$ values (10–40‰)[9]. In our mixing modelling, an average of $\delta^{18}O$ of 25‰ is assumed for the subducted Neo-Tethys oceanic sediments.

Gangdese juvenile arc melt is represented by the pre-collisional gabbros (110–70 Ma) with SiO_2 = 48–52 wt.% and Hf = 1.29 ppm (median value of 104 samples). Data are listed in **Supplementary Table 1**.

Gangdese ancient basement-derived melt is represented by the 365–340 Ma gneissic granite and two-mica granite in the Gangdese belt[13]. These rocks show geochemical signatures of S-type granite and underwent amphibolite-facies metamorphism under P-T conditions of 0.6–0.7 GPa and 690–740 °C at ~107 Ma[14]. Therefore, these rocks are considered to represent the ancient metasedimentary basement at a depth of the Gangdese middle crust.

Indian middle crust-derived melt is represented by the ~131 Ma rhyolite[15] and ~140 Ma A-type granite derived from anatexis of metapelitic rock under low pressure (< 0.5 GPa)[16]. These rocks are considered to represent metasedimentary rock-derived melt in the Indian middle crust, given that the northern margin of the Indian plate has a normal crustal thickness (~33 km) during the Early Cretaceous.

Indian upper crust-derived melt is represented by the ~14 Ma Himalayan leucogranite (sample 1G01)[17]. This sample is inferred to represent a pure metasedimentary rock-derived melt in the Indian upper crust, given that the continental crust of the northern Indian plate has been thickened to > 50 km during the Miocene.

The whole-rock Hf abundance and zircon Hf-O isotopic compositions of each end member for binary mixing modelling in **Fig. 4c-e** are given in **Supplementary Table 5**. Binary modelling shows the input of ~5 wt% of subducted oceanic sediment can explain the ~1‰ systematic increase in zircon $\delta^{18}O$, whereas 0–20 wt% of ancient Gangdese basement-derived melt can explain the pre-collisional samples (**Fig. 4c**). The input of 0–10 wt% of Indian middle crust-derived melt can account for the syn-collisional samples (**Fig. 4d**). Increased amounts of Indian middle to upper crust-derived melt (up to 10–40 wt%) may have been involved in the post-collisional samples (**Fig. 4e**).

Supplementary Figures

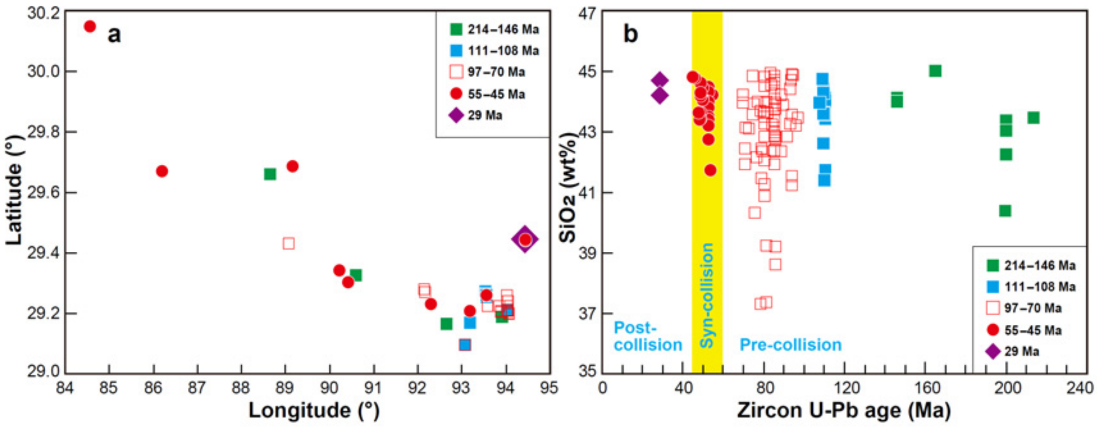

Supplementary Fig. 1 | Spatial and temporal distributions of ultramafic rocks (SiO_2 < 45 wt%) in the Gangdese belt. a Shows that the pre-collisional ultramafic rocks extend discretely for ~500 km along strike from longitude E89° to E94°. **b** Shows that the pre-collisional ultramafic rocks were mainly emplaced between 214 and 70 Ma, followed by ultramafic rocks formed during syn- and post-collision. Data are given in **Supplementary Table 1**.

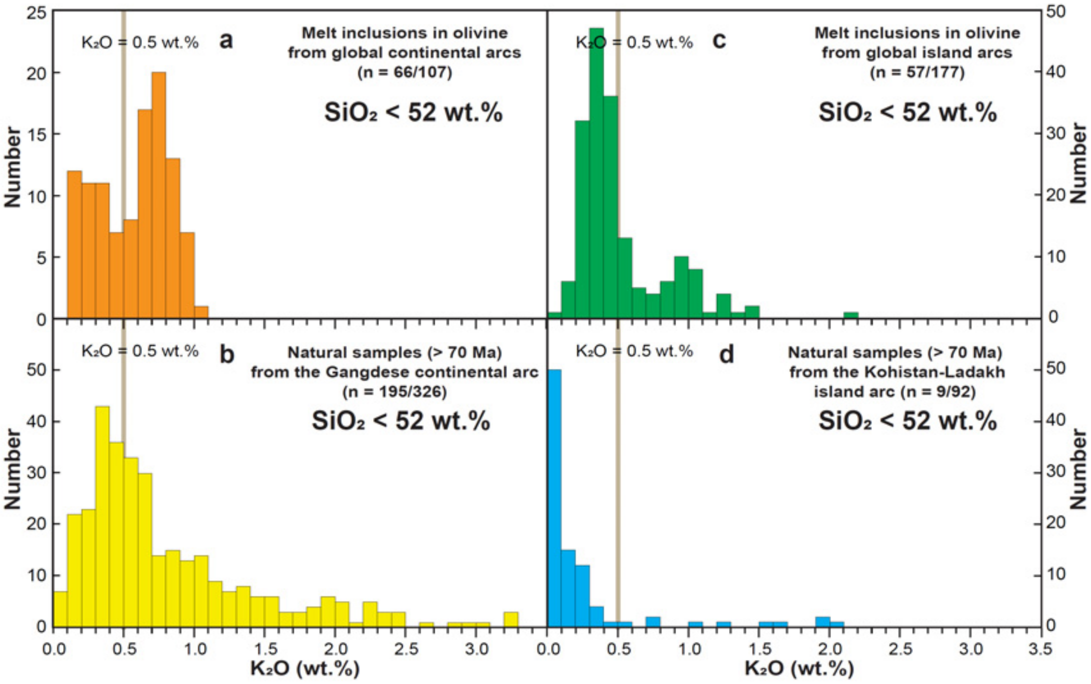

Supplementary Fig. 2 | Distributions of K_2O contents of samples from continental and island arcs. a K_2O contents of melt inclusions in olivine from tholeiitic/calc-alkaline volcanic rocks from global continental arcs (data from http://georoc.mpch-mainz.de.gwdg.de/georoc/) (**Supplementary Table 6**). **b** K_2O contents of natural samples (> 70 Ma) from the Gangdese continental arc (**Supplementary Table 7**). **c** K_2O contents of melt inclusions in olivine from tholeiitic/calc-alkaline volcanic rocks from global island arcs (data from http://georoc.mpch-mainz.de.gwdg.de/georoc/) (**Supplementary Table 6**). **d** K_2O contents of natural samples (> 70 Ma) from the Kohistan-Ladakh island arc (data from refs. 18, 19, and references therein) (**Supplementary Table 8**). Numerator and denominator indicate the number of samples with K_2O > 0.5 wt.% and the total numbet of samples, respectively.

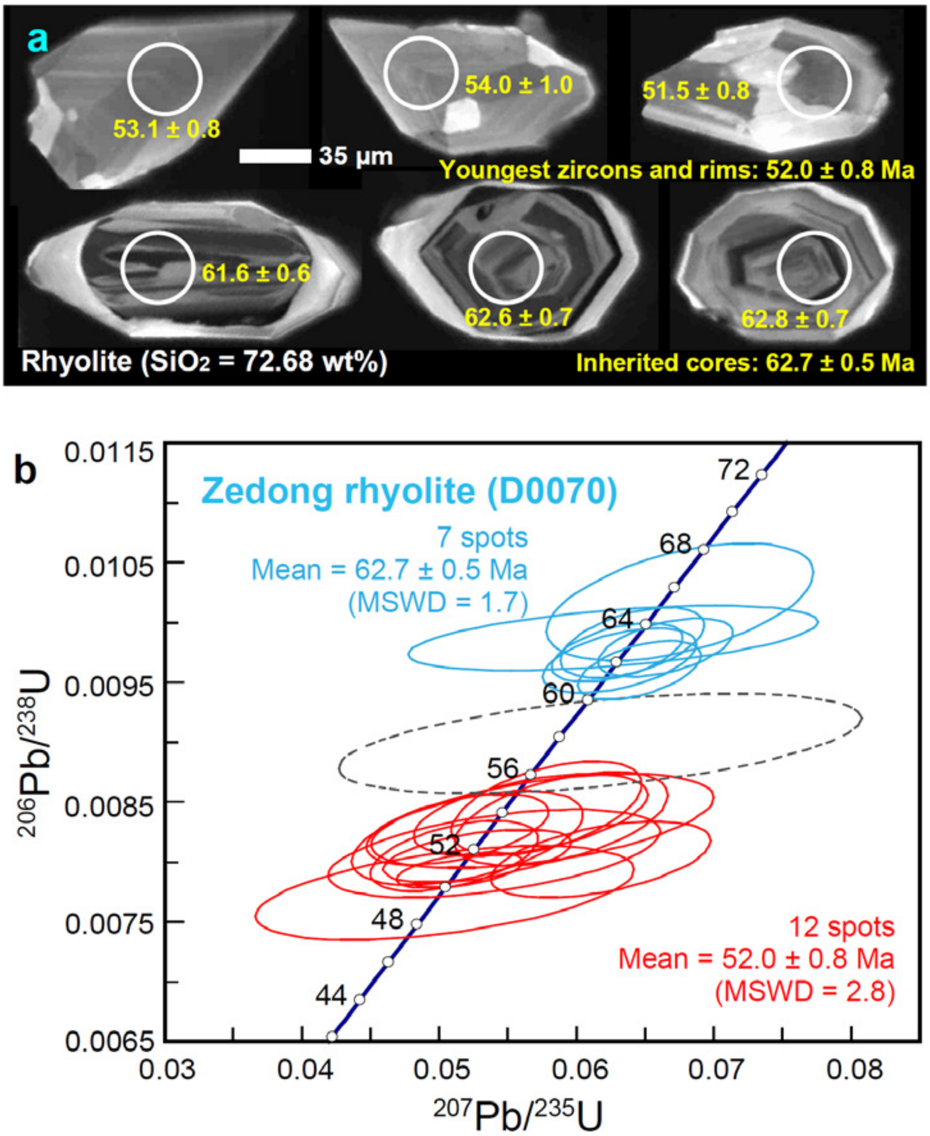

Supplementary Fig. 3 | Zircon evidence for the re-melting of older crustal rocks in the Gangdese belt. a Cathodoluminescence (CL) images of zircons from the Zedong rhyolite showing co-magmatic zircons (~52 Ma) and inherited cores (~63 Ma) with rims. **b** Concordia plots of the Zedong rhyolite. Data of dashed ellipses are not included in calculating weight mean age. Zircon U-Pb age data are provided in **Supplementary Table 2**.

Supplementary References

1. Zhang, L. L., Zhu, D. C., Wang, Q., Zhao, Z. D., Liu, D. & Xie, J. C. Late Cretaceous volcanic rocks in the Sangri area, southern Lhasa Terrane, Tibet: Evidence for oceanic ridge subduction. *Lithos* **326–327**, 144–157 (2019).
2. Jackson, S. E., Pearson, N. J., Griffin, W. L. & Belousova, E. A. The application of laser ablation-inductively coupled plasma-mass spectrometry to in situ U–Pb zircon geochronology. *Chem. Geol.* **211,** 47–69 (2004).
3. Slama, J., Kosler, J., Condon, D. J., Crowley, J. L., Gerdes, A., Hanchar, J. M. et al., 2008. Plesovice zircon - A new natural reference material for U–Pb and Hf isotopic microanalysis. *Chem. Geol.* **249**, 1–35 (2008).
4. Li, X., Tang, G., Gong, B., Yang, Y., Hou, K., Hu, Z., Li, Q., Liu, Y. & Li, W. Qinghu zircon: A working reference for microbeam analysis of U-Pb age and Hf and O isotopes. *Chin. Sci. Bull.* **58**, 4647–4654 (2013).
5. Tang, G. Q., Li, X. H., Li, Q. L., Liu, Y., Ling, X. X. & Yin, Q. Z. Deciphering the physical mechanism of the topography effect for oxygen isotope measurements using a CAMECA IMS-1280 SIMS. *J. Anal. Spectrom.* **30**, 950–956 (2015).
6. DePaolo, D.J. & Wasserburg, G.J. Petrogenetic mixing models and Nd-Sr isotopic patterns. *Geochim. Cosmochim. Acta* **43**, 615–627 (1979).
7. Elburg, M. A. & Foden, J. Geochemical response to varying tectonic settings: an example from southern Sulawesi (Indonesia). *Geochim. Cosmochim. Acta* **63**,1155–1172 (1999).
8. Jagoutz, O., Bouilhol, P., Schaltegger, U. & Müntener, O. The isotopic evolution of the Kohistan Ladakh arc from subduction initiation to continent arc collision. *Spec. Publ. - Geol. Soc. London*. **483**, 165–182 (2019).
9. Valley, J. W., Lackey, J. S., Cavosie, A. J., Clechenko, C. C., Spicuzza, M. J., Basei, M. A. S., Bindeman, I. N., Ferreira, V. P., Sial, A. N., King, E. M., Peck, W. H., Sinha, A. K. & Wei, C. S. 4.4 billion years of crustal maturation: oxygen isotope ratios of magmatic zircon. *Contrib. Mineral. Petrol.* **150**, 561–580 (2005).
10. Lackey, J. S., Valley, J. W. & Saleeby, J. B. Supracrustal input to magmas in the deep crust of Sierra Nevada batholith: evidence from high-δ18O zircon. Earth *Planet. Sci. Lett.* **235**, 315–330 (2005).
11. Zhang, L.L., Liu, C.Z., Wu, F.Y., Zhang, C., Ji, W.Q., Wang, J.G., Sr-Nd-Hf isotopes of the intrusive rocks in the Cretaceous Xigaze ophiolite, southern Tibet: Constraints on its formation setting. *Lithos* **258–259**, 133–148 (2016).
12. Chauvel, C., Lewin, E., Carpentier, M., Arndt, N.T., Marini, J.C., Role of recycled oceanic basalt and sediment in generating the Hf-Nd mantle array. *Nat. Geosci.* **1**, 64–67 (2008).
13. Ma, L., Kerr, A.C., Wang, Q., Jiang, Z.Q., Tang, G.J., Yang, J.H., Xia, X.P., Hu, W.L., Yang, Z.Y., Sun, P., Nature and evolution of crust in southern Lhasa, Tibet: transformation from microcontinent to juvenile terrane. *J. Geophys. Res., Solid Earth* **124**, 6452–6474 (2019).
14. Dong, X., Zhang, Z.M., Liu, F., He, Z.Y., Lin, Y.H., Late Paleozoic intrusive rocks from the southeastern Lhasa terrane, Tibetan Plateau, and their Late Mesozoic metamorphism and tectonic implications. *Lithos* **198–199**, 249–262 (2014).
15. Xia, Y., Zhu, D.C., Zhao, Z.D., Wang, Q., Yuan, S.H., Chen, Y., Mo, X.X., Whole-rock geochemistry and zircon Hf isotope of the OIB-type mafic rocks from the Comei large igneous province in southeastern Tibet. *Acta Petrologica Sinica* **28**, 1588–1602 (2012).

16. Ma, L., Kerr, A.C., Wang, Q., Jiang, Z.Q., Hu, W.L., Early Cretaceous (~140 Ma) aluminous A-type granites in the Tethyan Himalaya, Tibet: Products of crust-mantle interaction during lithospheric extension. *Lithos* **300–301**, 212–226 (2018).
17. Hopkinson, T.N., Harris, N.B.W., Warren, C.J., Spencer, C.J., Roberts, N.M.W., Horstwood, M.S.A., Parrish, R.R., EIMF, The identification and significance of pure sediment-derived granites. *Earth Planet. Sci. Lett.* **467**, 57–63 (2017).
18. Dhuime, B., Bosch, D., Bodinier, J.L., Garrido, C.J., Bruguier, O., Hussain, S.S., Dawood, H., Multistage evolution of the Jijal ultramafic–mafic complex (Kohistan, N Pakistan): Implications for building the roots of island arcs. *Earth Planet. Sci. Lett.* **261**, 179–200 (2007).
19. Jagoutz, O., Müntener, O., Schmidt, M.W., Burg, J.P., The roles of flux- and decompression melting and their respective fractionation lines for continental crust formation: Evidence from the Kohistan arc. *Earth Planet. Sci. Lett.* **303**, 25–36 (2011).
