# Supplementary material for: Interplay between oceanic subduction and continental collision in building continental crust
Source: Nat Commun. 2022 Nov 21;13:7141. doi: 10.1038/s41467-022-34826-0 (PMC9681875; doi:10.1038/s41467-022-34826-0)
Supplement: Supplementary file 3 — Description of Additional Supplementary files [file 41467_2022_34826_MOESM3_ESM.pdf]

Description of Additional Supplementary Files

File name: Supplementary Table 1
Description: Whole-rock geochemical indices of samples from the Gangdese Batholith

File name: Supplementary Table 2
Description: Zircon U-Pb age data of samples from the Gangdese Batholith

File name: Supplementary Table 3
Description: Mineral compositions of samples from the Gangdese Batholith

File name: Supplementary Table 4
Description: Zircon Hf-O isotopic data of samples from the Gangdese Batholith

File name: Supplementary Table 5
Description: Whole-rock Hf abundance and isotopic compositions of zircons for binary mixing modelling

File name: Supplementary Table 6
Description: K2O contents of melt inclusions in olivine from tholeiitic/calc-alkaline volcanic rocks from global continental and island arcs

File name: Supplementary Table 7
Description: K2O contents of natural samples (> 70 Ma) from the Gangdese continental arc

File name: Supplementary Table 8
Description: K2O contents of natural samples (> 70 Ma) from the Kohistan-Ladakh Island arc
